# A 3'UTR Polymorphism of IL-6R Is Associated with Chinese Pediatric Tuberculosis

**DOI:** 10.1155/2014/483759

**Published:** 2014-03-19

**Authors:** Chen Shen, Hui Qi, Lin Sun, Jing Xiao, Qing-qin Yin, Wei-wei Jiao, Xi-rong Wu, Jian-ling Tian, Rui Han, A-dong Shen

**Affiliations:** Key Laboratory of Major Diseases in Children and National, Key Discipline of Pediatrics, (Capital Medical University), Ministry of Education, Beijing Pediatric Research Institute, Beijing Children's Hospital, Capital Medical University, No. 56 Nan-li-shi Road, Beijing 100045, China

## Abstract

*Background*. IL-6 is a proinflammatory cytokine that plays a critical role in host defense against tuberculosis (TB). Genetic polymorphisms of IL-6 and its receptor IL-6R had been discussed in adult TB recently. However, their role in pediatric TB is still unclear. Due to the obvious differences in TB pathophysiology in children, which may also reflect differences in genetic background, further association studies in pediatric populations are needed. *Methods*. A case-control study was carried out in a Chinese pediatric population including 353 TB patients and 400 healthy controls. Tag-SNPs of IL-6 and IL-6R genes were selected by Haploview software, genotyped using MassArray, and analyzed statistically. *Results*. One polymorphism, rs2229238, in the 3'UTR region of IL-6R was observed to be associated with increased resistance to TB (adjusted *P* = 0.03). The rs2229238 T allele contributed to a reduced risk to TB in recessive heritable model (OR, 0.53; 95% CI, 0.35–0.78). *Conclusions*. By tag-SNP genotyping based case-control study, we identified a genetic polymorphism in the IL-6R 3'UTR that regulates host resistance to pediatric TB in a Chinese population.

## 1. Introduction

Caused by * Mycobacterium tuberculosis* (*M. tuberculosis*) infection, tuberculosis (TB) remains to be a major global public health concern. In China, the prevalence of TB is 1.08‰ [1] in adults and 0.918‰ [2] in children. Host genetic factors play an essential role in determining TB susceptibility or resistance [3]. Compared with adults, children present a special risk group for TB due to rapid progression, significant morbidity, and mortality [4–6]; thus, the genetic background of pediatric TB might be quite different from adult TB. While most of association studies have been focused on TB in adults, childhood TB has been relatively neglected. Studies of TB genetics in well-defined pediatric populations are therefore needed.

As a major proinflammatory cytokine, Interleukin-6 (IL-6) takes part in the protection from pathogens infection. By binding to Interleukin-6 receptor (IL-6R), IL-6 triggers the intracellular signaling cascade that leads to inflammatory responses [7]. Elevated IL-6 from bronchoalveolar lavage cells seemed to be biomarkers of noncavitary TB [8]. An association study of genetic polymorphisms of IL-6 and its receptor IL-6R had recently been conducted in a group of Chinese population, suggesting a promoter polymorphism in IL-6 associated with adult TB [9]. However, the potential associations and molecular roles of IL-6 and IL-6R in regulating susceptibility or resistance to pediatric TB are still undiscovered. As childhood TB seems to have more genetic predisposition [5], we thus tried to discuss the associations of IL-6 and IL-6R with Chinese pediatric TB by single polymorphism genotyping (SNP) based case-control study.

## 2. Materials and Methods

### 2.1. Ethics Statement

Clinical investigation had been conducted according to the principles expressed in the Declaration of Helsinki. This research has been approved by the Ethics Committee of Beijing Children's Hospital. Written informed consent was obtained from all the participants or their guardians in this research.

### 2.2. Study Sample

All the participants involved in this research were Han ethnicity. The pediatric TB patients (*n* = 353) were newly diagnosed to be pulmonary TB (PTB, pathological changes limited to lung) or extrapulmonary TB (EPTB, pathological changes involving other tissues) by at least two experienced pediatricians in Beijing Children's Hospital according to the pediatric TB clinical diagnosis standard [10–13]. The diagnostic criteria of pediatric TB had been described in our previous paper [14].

Participants of the control group (*n* = 400) were recruited among those admitted to Beijing Children's Hospital for physical examination. All of them had negative tuberculin PPD skin-test results (<5 mm) and no history of TB or HIV infection and were matched with TB cases for age, sex, and ethnicity.

### 2.3. DNA Extraction and Genotyping

Tag-SNPs of IL-6 and IL-6R were selected following data release from Phase II of the International HapMap project [15]. Sample based genotypes were downloaded for all variants in genomic regions including from 5,000 bp 5-prime upstream to 5000 bp 3-prime downstream of IL-6 and IL-6R independently.

Since the study populations under investigation were from the Chinese population, downloaded genotypes were restricted to those for the Han Chinese in Beijing, China (CHB) population (http://hapmap.ncbi.nlm.nih.gov). Tag-SNPs were selected using a pairwise tagging algorithm by Haploview software (available at http://www.broadinstitute.org/haploview), with a correlation coefficient (r2) exceeding 0.8 for all downloaded SNPs with minor allele frequency (MAF) >5% [16]. Because the tag-SNP probabilities were discrete, accordingly, functional ranking of tag-SNPs with the same probability was used.

Blood samples from all participants were collected and stored at −20°C. Genomic DNA was extracted from peripheral leukocytes by using a Genomic DNA Extraction kit (QIAamp DNA Blood Mini Kit; Qiagen, Hilden, Germany). MassArray (Sequenom, USA) was used for genotyping selected tag-SNPs (Gabriel et al., 2009), and this assay was accomplished by Bio Miao Biological Technology (Beijing, China). The primers were designed using iPLEX GOLD (Sequenom, USA) [17].

### 2.4. Statistical Analysis

Statistical analysis was carried out using the Statistical Package for SNP Stats software (http://bioinfo.iconcologia.net/snpstats/start.htm) and PLINK software (version 1.07) (http://pngu.mgh.harvard.edu/~purcell/plink/). The Hardy-Weinberg equilibrium (HWE) was performed using the SHEsis program (http://analysis.bio-x.cn/myAnalysis.php). Chi-square tests were used for unordered categorical variable. Significant differences were indicated by a *P* value <0.05. Bonferroni correction for multiple testing was used. Adjusted odds ratio (AOR) and 95% confidence interval (CI) were calculated by logistic regression analysis.

## 3. Results

### 3.1. Patients and Controls

The mean age was SD, 4.7; range, 2 months–16.5 years for TB patients and 6.1 years (SD, 3.8; range, 3 months–17 years) for the non-TB control subjects. TB cases in our research include 156 (44.2%) PTB patients and 197 (55.8%) EPTB patients. Here, we also defined severe TB (SeTB) to be patients with disseminated TB (DTB) and tuberculosis meningitis (TBM), which both belonged to EPTB but presented severe clinical manifestations and usually poor outcomes. SeTB was identified in 81.2% of EPTB cases (160/197). Detailed characteristics of study population are shown in Table 1.

### 3.2. Selected Tag-SNPs

To conduct the association study, gene polymorphisms were selected using the criteria mentioned above in Section 2. Thus, two IL-6 tag-SNPs and ten IL-6R tag-SNPs were finally selected for genotyping. Of the 2 selected tag-SNPs of IL-6, one existed in the near 5′ of IL-6 (rs17147230) and another in the IL-6 promoter region (rs1800796). The rs1800796 SNP is located in the IL-6 promoter and has been thought to be associated with adult TB in Chinese population recently [9]. The tag-SNP rs17147230 was thought to be functionally associated with plasma adrenomedullin levels by one research group [18]. Meanwhile, 10 tag-SNPs of IL-6R were included in our genotyping. Of the 10 tag selected SNPs of IL-6R, one (rs3887104) is located in the promoter region, one (rs4845617) in the 5-prime untranslated region (5′UTR), three (rs7411976, rs4845618, rs4845626) in the intron region, two (rs2228145, rs8192284) present to be missense SNPs in coding region and other three (rs2229238, rs4072391, rs3828078) in 3-prime untranslated region (3′UTR).

### 3.3. Genotyping and Genetic Analysis

Total selected 12 single-nucleotide polymorphisms (SNPs) were genotyped. One SNP (rs8192284) data was wiped off for nonspecific amplification, according to the clustering performance. The rs4072391 SNP was not in Hardy-Weinberg equilibrium (HWE, *P* = 0.013) in the control group and thus was ruled out for further analysis. The rest 10 tag-SNPs were in Hardy-Weinberg equilibrium (HWE, *P* > 0.05) in the control group, which were sent to further analysis. The genotyping results of detected SNPs are summarized in Table 2.

Genetic association of rs2229238, an IL-6R 3′UTR SNP, with TB disease was observed after an application of the Bonferroni correction for multiple testing. The frequency of T allele in TB group was significantly lower than that in control group (OR: 0.57, 95% CI: 0.39–0.83, Bonferroni *P* = 0.033). Genotypic distribution of rs2229238 also revealed significant difference between TB group and control group (Bonferroni *P* = 0.05). Further, a 2 × 2*χ* ^2^ test was used by combining different genotype combinations to test dominant (TC + TT versus CC) and recessive (TT versus CC + TC) models of inheritance. The OR for the T allele of rs2229238 as a possible risk factor was 0.53 (95% CI: 0.35–0.78, *P* = 0.001) under a dominant model and 1.13 (95% CI: 0.16–8.07, *P* = 0.9) under a recessive model (Table 3(a)). Thus, a Mendelian dominant trait of T allele was accepted for the inheritance pattern.

To further examine associations of the rs2229238 polymorphism genotypes with different clinical forms of TB, we compared PTB and EPTB subgroups with control group independently (Table 3(b)). Both the frequencies of rs2229238 T allele and rs2229238 TC + TT combined genotypes decrease progressively from controls to PTBs, then to EPTBs. Significant differences of rs2229238 allele were found between EPTB and controls (*P* = 0.003), but not between PTB and controls (*P* = 0.109). The frequency of TC + TT genotypes (T allele carrying) was significantly lower in EPTB patients than that in controls (OR: 0.44, 95% CI: 0.26–0.74, *P* = 0.0035), while the frequency of TC + TT genotypes was not significantly lower in the PTB group than that in controls (OR: 0.64, 95% CI: 0.39–1.06, *P* = 0.073).

## 4. Discussion

Unlike adults, children present rapid progression from a recent infection towards disease and are vulnerable to severe disease and death [4–6]. Certain pediatric TB reflects Mendelian predispositions, while adult TB seems to be more complex for genetic predisposition [5]. Studying the effects of the candidate susceptibility genes on pediatric TB may aid in the establishment of more efficient prevention of TB spread.

Interleukin-6 (IL-6) is a pleiotropic cytokine with important roles in immunoregulation [7]. But the role of IL-6 in limiting* M. tuberculosis* infection is still under discussion. Recently, Zhang et al. [9] proved in a Chinese Han population that an IL-6 promoter variation, which functionally downregulated IL-6 producing, was protective against TB. Elevated IL-6 from bronchoalveolar lavage cells seemed to be a biomarker of noncavitary TB [8]. In mice, increased IL-6 level was found to be correlated with TB progression [19]. IL-6 could downregulate the microbicidal activity of macrophage [20, 21]. But Ladel et al. [22] believed that IL-6 could play critical role in host resistance to* M. tuberculosis* infection that IL-6 deficient mice had increased bacterial loads when infected by* M. tuberculosis*. Some other researchers demonstrated that although IL-6 could induce early interferon-gamma production in the infected lung and the absence of IL-6 led to a delay in the induction of protective immunity with a subsequent early increase in bacterial load, however, the absence did not affect the induction of normal protective memory responses, which means IL-6 might not be required for generation of specific immunity to* M. tuberculosis* infection [23].

As the receptor of IL-6, IL-6R plays an important role in IL-6 signaling cascade [7]. IL-6 acts through binding specifically to the IL-6R to form a complex, and then this complex binds to the ubiquitousgp130 subunit to trigger intracellular signaling. IL-6R could be expressed both in membrane-bound form and a cleaved soluble form of IL-6R (sIL-6R). Increased level of sIL-6R has been reported in immune-related diseases, such as diabetes and allergic asthma [24, 25]. IL-6R controls lung CD4^+^CD25^+^Foxp3^+^ regulatory T cells (Treg) development, with sIL-6R regulating Th2 cell functions in CD4^+^CD25^−^ effectors T cells lacking mIL-6R and mIL-6R controlling cell fate at the beginning of T-cell differentiation by directing CD4^+^ naive cells toward Th2 pathways and inhibiting Treg differentiation [26]. The imbalance between effectors T cells and Tregs was also thought to play an important role in TB etiology [27].

In this study, we try to illustrate the association of IL-6 and its receptor IL-6R with pediatric TB by tag-SNP genotyping. A tag single-nucleotide polymorphism (SNP) is representative SNP in a region of the genome with high linkage disequilibrium (the nonrandom association of alleles at two or more loci). It is possible to identify genetic variation without genotyping each SNP in a chromosomal region. Tag-SNPs are useful in gene association studies. Previously, rs1800796 (−572 C/G) of IL-6 was detected to be associated with adult TB and the regulatory effects of this SNP on IL-6 production in plasma and CD14^+^ monocyte cultures stimulated with a* M. tuberculosis *product were also conformed [9]. CD14^+^ monocytes with rs1800796 GG genotype produced less IL-6 in response to* M. tuberculosis* 19 kDa lipoprotein than those with CC or CG genotype. To our surprise, we did not find this potential association of rs1800796 with TB in our pediatric group. This might depend on the genetic and clinical differences between TB on set childhood and adulthood as we have described above. Another possibility of this inconformity might be due to different genetic backgrounds between two populations: our samples are mainly from North China, while Zhang's samples are from South China. We noticed that the G allele frequencies between these two researches are obviously different. In Zhang's research, the allele frequencies of rs1800796 G are 26.1% in controls and 21.3% in cases, while in our research, these frequencies are 34.4% and 33.7% independently.

Our results indicate that a 3′UTR polymorphism variation within IL-6R, rs2229238, contributes to pediatric TB resistance. The data showed that both the frequencies of rs2229238T allele and rs2229238 TC + TT combined genotypes decrease progressively from control to PTB, then to EPTB. As we know, to children, most of EPTB are developed from PTB, which means the rs2229238 SNP might contribute to TB disease progression. Unlike adults, pediatric EPTB usually presents more serious and complicated clinical symptoms and also poor outcomes. In our research, SeTB, which presents severe clinical manifestations, was identified in 81.2% of EPTB cases. Thus, rs2229238 T allele might protect children from both TB on set and disease progression.

Previously, in analyzing the potential genetic associations between four polymorphisms of IL-6R and atherosclerotic lipid profiles among young adolescents in Taiwan, Chu et al. [28] found the IL-6R rs2229238 C/T variants being associated with dyslipidemia in girls. By screening the association of eleven IL-6R gene variants with type 2 diabetes in Northern European Caucasian and African American ethnic groups, Wang et al. [24] identified the rs2229238 polymorphism in the 3′UTR showing a trend to an association with type 2 diabetes in a Caucasian population (*P* = 0.055). But no association study of rs2229238 C/T variants with pediatric TB has been confirmed before.

One weakness of this study is that we did not investigate how rs2229238 C/T was functionally involved in the susceptibility and development of TB. In the future, we could try to do some functional studies, for instance, to discover whether different alleles of this SNP differ in IL-6R expression.

## 5. Conclusion

In conclusion, we discussed the associations of IL-6 and IL-6R SNPs with TB in a Chinese pediatric population and identified rs2229238 T allele presenting a protective role in both pediatric TB on set and disease progression. Additional studies are warranted to test out result in other pediatric populations.

## Figures and Tables

**Table 1:** Demographic characteristics of study population.

Characteristic	TB (*n* = 353)	Control (*n* = 400)	*P*
Gender			
Male, *n* (%)	223 (63.2)	236 (59.0)	0.385^a^
Female, *n* (%)	130 (36.8)	164 (41.0)	
Age			
Mean year (SD)	5.7 (4.7)	6.1 (3.8)	0.144^a^
TB type			
PTB (%)	156 (44.2)		
EPTB (%)	197 (55.8)		
DTB	71		
TBM	90		
Abdominal TB	21		
TBL	12		
Bone or joint TB	2		
Other EPTB	1		

TB: tuberculosis; PTB: pulmonary tuberculosis; EPTB: extrapulmonary TB; DTB: disseminated TB; TBM: tuberculous meningitis; TBL: tuberculous lymphadenitis. ^a^ *P* value was calculated by *t* test.

**Table 2:** The genotyping results of SNPs in IL-6 and IL-6R.

Gene	Position	Rs No.	Allele/genotype	TB (*n*, %)	Control (*n*, %)	OR	*P*	Bonf *P*
IL6	5 near	rs17147230	A	334, 47.3	388, 48.7	0.94 [0.77–1.56]	0.58	5.79
			T	372, 52.7	408, 51.3			
			AA	83, 23.5	94, 23.6		0.67	6.70
			TA	168, 47.6	200, 50.2			
			TT	102, 28.9	104, 26.1			
	Promoter	rs1800796	G	243, 33.7	269, 34.4	1.03 [0.83–1.28]	0.77	7.72
			C	463, 66.3	529, 65.6			
			GG	43, 12.2	49, 12.3		0.90	9.00
			GC	157, 44.5	171, 42.9			
			CC	153, 43.3	179, 44.9			

IL-6R	Promoter	rs3887104	A	65, 9.4	56, 7.2	1.33 [0.92–1.93]	0.13	1.34
			G	629, 90.6	720, 92.8			
			AA	3, 0.9	0, 0		0.06	0.63
			AG	59, 17.0	56, 4.4			
			GG	285, 82.1	332, 85.6			
	5 UTR	rs4845617	A	370, 47.4	322, 48.6	0.95 [0.77–1.17]	0.65	6.48
			G	358, 52.6	392, 51.4			
			AA	78, 22.9	90, 23.6		0.87	8.70
			AG	166, 48.8	190, 49.9			
			GG	96, 28.2	101, 26.5			
	Intron	rs7411976	C	39, 5.6	34, 4.4	1.27 [0.79–2.04]	0.32	3.19
			A	661, 94.4	732, 95.6			
			CC	2, 0.6	0, 0		0.20	2.00
			CA	35, 10	34, 8.9			
			AA	313, 89.4	349, 91.1			
	Intron	rs4845618	G	320, 46.1	342, 44.0	1.09 [0.89–1.34]	0.41	4.08
			T	374, 53.9	436, 56.0			
			GG	77, 22.2	73, 18.8		0.51	5.10
			GT	166, 47.8	196, 50.4			
			TT	104, 30	120, 30.9			
	Intron	rs4845626	T	57, 8.1	71, 8.9	0.90 [0.63–1.30]	0.58	5.78
			G	649, 92.9	729, 91.1			
			TT	4, 1.1	1, 0.2		0.15	1.50
			TG	49, 13.9	69, 17.2			
			GG	300, 85.0	330, 82.5			
	Missense	rs2228145	C	290, 41.7	350, 46.5	0.82 [0.67–1.01]	0.06	0.62
			A	406, 58.3	402, 53.5			
			CC	60, 17.2	81, 21.5		0.17	1.70
			CA	170, 48.9	188, 50.0			
			AA	118, 33.9	107, 28.5			
	3 UTR	rs2229238	T	46, 6.5	87, 10.9	0.57 [0.39–0.83]	0.003	0.03
			C	660, 93.5	711, 89.1			
			TT	2, 0.6	2, 0.5		0.004	0.04
			TC	42, 11.9	83, 20.8			
			CC	309, 87.5	314, 78.7			
	3 UTR	rs3828078	A	43, 6.1	34, 4.3	1.46 [0.92–2.31]	0.11	1.08
			G	663, 93.9	764, 95.7			
			AA	0, 0	2, 0.5		0.03	0.29
			AG	43, 12.2	30, 7.5			
			GG	310, 87.8	367, 92.0			

Logistic regression analyses were used for calculating OR (95% CI: confidence interval). Value was determined by Fisher's exact test.

**Table:** (a) Analysis of the inheritance models of IL-6R rs2229238 polymorphism in the combined samples

Model	Genotype	TB (*n*, %)	Control (*n*, %)	*P* value	OR (95% CI)
Dominant	CC	309, 87.5	314, 78.7	0.001	0.53 (0.35–0.78)
	TC + TT	85, 21.3	44, 12.5		

Recessive	CC + TC	351, 99.4	397, 99.5	0.9	1.13 (0.16–8.07)
	TT	2, 0.6	2, 0.5		

**Table:** (b) Comparison of the genotypes and alleles distribution in different patient subgroups stratified by diagnosis

Subjects (*n*)*	Genotype (*n*, %)		*P* ^1^	OR^1^ (95% CI)	Allele (*n*, %)		*P* ^2^	OR^2^ (95% CI)
	TC + TT	CC			T	C		
Control (400)	85, 21.3	314, 78.7	—		87, 10.9	711, 89.1	—	
TB (273)	44, 12.5	309, 87.5	0.004	0.53 (0.35–0.78)	46, 6.5	660, 93.5	0.003	0.57 (0.39–0.83)
PTB (156)	23, 14.7	133, 85.3	0.073	0.64 (0.39–1.06)	24, 7.7	288, 92.3	0.109	0.68 (0.42–1.09)
EPTB (197)	21, 10.7	176, 89.3	0.004	0.44 (0.26–0.74)	22, 5.6	372, 94.4	0.003	0.48 (0.30–0.78)

*PTB: pulmonary tuberculosis; EPTB: extrapulmonary. ^1^ *P* value and OR (95% CI) of 2 × 2  *χ* ^2^ test for dominant inheritance of T allele; ^2^ *P* value and OR (95% CI) of 2 × 2  *χ* ^2^ tests for allele. ^1^OR (95% CI) for dominant inheritance of T allele. ^2^OR (95% CI).
